# Aromatase Inhibitors—Induced Musculoskeletal Disorders: Current Knowledge on Clinical and Molecular Aspects

**DOI:** 10.3390/ijms21165625

**Published:** 2020-08-06

**Authors:** Sara Tenti, Pierpaolo Correale, Sara Cheleschi, Antonella Fioravanti, Luigi Pirtoli

**Affiliations:** 1 Rheumatology Unit, Department of Medicine, Surgery and Neuroscience, Azienda Ospedaliera Universitaria Senese, Policlinico Le Scotte, Viale Bracci 1, 53100 Siena, Italy; sara_tenti@hotmail.it (S.T.); fioravanti7@virgilio.it (A.F.); 2 Medical Oncology Unit, Grand Metropolitan Hospital “Bianchi-Melacrino-Morelli”, 89121 Reggio Calabria, Italy; correalep@yahoo.it; 3 Sbarro Institute for Cancer Research and Molecular Medicine-Center for Biotechnology, Department of Biology, College of Science and Technology, Temple University, Philadelphia, PA 19122, USA; luigipirtoli@gmail.com

**Keywords:** aromatase inhibitors, breast cancer, aromatase inhibitors-associated arthralgia, autoimmune rheumatic diseases, musculoskeletal disorders, hormonal anti-estrogen therapy, endocrine therapy

## Abstract

Aromatase inhibitors (AIs) have radically changed the prognosis of hormone receptor positive breast cancer (BC) in post-menopausal women, and are a mainstay of the adjuvant therapy for BC after surgery in place of, or following, Tamoxifen. However, AIs aren’t side effect-free; frequent adverse events involve the musculoskeletal system, in the form of bone loss, AI-associated arthralgia (AIA) syndrome and autoimmune rheumatic diseases. In this narrative review, we reported the main clinical features of these three detrimental conditions, their influence on therapy adherence, the possible underlying molecular mechanisms and the available pharmacological and non-pharmacological treatments. The best-known form is the AIs-induced osteoporosis, whose molecular pathway and therapeutic possibilities were extensively investigated in the last decade. AIA syndrome is a high prevalent joint pain disorder which often determines a premature discontinuation of the therapy. Several points still need to be clarified, as a universally accepted diagnostic definition, the pathogenetic mechanisms and satisfactory management strategies. The association of AIs therapy with autoimmune diseases is of the utmost interest. The related literature has been recently expanded, but many issues remain to be explored, the first being the molecular mechanisms.

## 1. Introduction

Hormonal manipulation represents the major therapeutic approach for women with breast cancer (BC) expressing estrogen receptors (ER). Particularly, the molecular pathologic pattern more sensitive to this kind of therapy is characterized by a high expression of ER, +/− Progesterone receptors (PR) expression, without HER2/neu growth factor receptors expression (Luminal A subtype), and accounts for 50% of cases. On the contrary, the Luminal B subtype, representing 15–20% of the cases and consisting of ≥20% ER expression, +/− PR expression, and variable positivity of the HER2/neu oncoprotein, is less responsive to hormone therapy [1,2]. The anti-estrogen hormonal therapy is based on the use, for at least five years, of ER agonists/antagonists, as Tamoxifen and of third generation aromatase inhibitors (AIs), as Anastrozole, Letrozole, and Exemestane. Both classes of drugs present a manageable toxicological profile, however, short- and long-term adverse events have been frequently reported, and deserve to be taken into consideration for the choice of specific treatment of these patients, who need to continue the cures for many years, in order to achieve a very long survival [3].

Tamoxifen is a selective estrogen receptor modulator (SERM), which partially competes with estrogens binding to ER, and it has been indicated both in pre- and post-menopausal women and males with hormone sensitive tumors for more than forty years. AIs, on the other hand, represent a relatively new generation of drugs for post-menopausal women with hormone sensitive BC, that act through inhibition of the aromatase. This enzyme converts androgens to estrogens and therefore its inhibition may empower estrogen deprivation related to the spontaneous menopausal condition, or in combination with luteinizing hormone-releasing hormone (LHRH) agonists [2]. In this context, the introduction of AIs has significantly impacted on the management of BC considering their ability to prolong patients’ survival and decrease the rate of tumor recurrence [4]. Thus, they have become the standard-of-care in post-menopausal women with ER-positive BC in the adjuvant setting. Particularly, AIs are recommended, for at least five years after the surgery, in place of, or following 2 to 3 years of Tamoxifen (sequential strategy), or at the end of Tamoxifen therapy (extended strategy) [5]. Furthermore, recent evidence suggests that the use of AIs in coordination with ovarian suppression (which can be obtained through three methods, as ovariectomy, ovarian radiotherapy and drug castration) can represent a new adjuvant treatment option also for pre-menopausal women with hormone receptor-positive BC, reducing the risk of recurrence [6].

Several reports suggest that AIs present a more favorable risk-benefit profile, compared to Tamoxifen, with a lower incidence of life-threatening adverse events, including thromboembolic episodes and occurrence of endometrial cancer, other than a better antitumor efficacy [7].

More recently, selective estrogen receptor down-regulators (SERDs), such as Fulvestrant, have entered in the treatment of hormone sensitive BC patients. These agents act as pure receptor antagonists with high affinity, and are able to induce downstream ER pathway inhibition and degradation of SERD-ER complex. Randomized clinical trials (RCTs) did not prove evidence that Fulvestrant exerts greater efficacy over Tamoxifen or AIs in the adjuvant setting, however, compared with these drugs, its use has been associated with a prolonged progression-free survival in patients with advanced and metastatic disease, where it is currently recommended, as first line treatment or as salvage therapy upon failure of Tamoxifen or AIs [8,9].

In general, hormonal manipulation remains a safe and promising treatment for adjuvant and metastatic hormone sensitive BC, with AIs presenting a greater antitumor efficacy and better safety profile, and their use is encouraged worldwide over cytotoxic chemotherapy or other antitumor means [3]. Although well tolerated, AIs are not completely free of adverse events; in this regard, a number of reports focused on their implication in the pathogenesis of several metabolic and immunological disorders that may have significant impact on the patients’ quality of life and treatment compliance. AIs, in fact, may enhance early menopausal systemic complications including, bone mineral density (BMD) decline, and musculoskeletal symptoms [4]. Furthermore, they can give rise to AI-associated arthralgia (AIA), characterized by symmetrical joint pain, mostly affecting hands, wrists and knees, whose symptoms relief immediately happens after AIs are discontinued; on the contrary, symptoms exacerbation occurs as soon as AIs are re-introduced [10]. The pathophysiology of AIA is still controversial and similarly, its impact on patients’ treatment compliance and quality of life is not clear yet. Besides, there are no treatments recommended for the management of this iatrogenic disease. Moreover, AIs therapies have been associated to the occurrence of autoimmune disorders, including rheumatoid arthritis (RA), spondyloarthropaty (SpA), Sjogren syndrome (SjS), systemic erythematosus lupus (SLE), systemic sclerosis (SS), anti-synthetase antibody syndrome (ASAS) and antiphospholipid syndrome (APS) [11]. Moreover, in this case, conclusive evidence concerning the pathogenesis of these conditions, as well as their incidence, prevalence, and time of onset, is still missing.

In this light, our narrative review focused on the current information concerning metabolic, immunological and non-immunological musculoskeletal adverse events related to AIs treatment for post-menopausal BC women. The following databases were searched from 1970 until 2020: Medline via PubMed, Cochrane Library and Web of Science with the MeSH terms of “Aromatase Inhibitors”, “Breast Cancer”, “Aromatase Inhibitors-associated arthralgia”, “autoimmune rheumatic diseases”, “musculoskeletal disorders”, “hormonal anti-estrogen therapy”, “endocrine therapy”.

## 2. Aromatase Inhibitors: Development and Pharmacology

The discovery of the role of aromatase, the critical enzyme for estrogen synthesis, has represented the first step for the research of specific inhibitors in the field of treatment of hormone sensitive BC, as an alternative to the classical SERMs, like Tamoxifen and/or LHRH agonists or ovariectomy. In this context, AIs development started in the early 1970s, when researchers recognized that aromatase could represent a therapeutic target in hormone-dependent BC [12], and several molecules with different biological properties were identified, and classified according to their specificity in inhibiting aromatase [13]. Aminoglutethimide, a drug introduced as an anti-convulsant in 1966, was the first drug approved by Food and Drug Administration (FDA) for BC treatment in post-menopausal women. This pharmacological agent showed an objective response rate of 20–40% in metastatic BC patients, but was unable to show superiority in terms of benefit and survival, over Tamoxifen [14]. Furthermore, Aminoglutethimide induced the additional, not selective inhibition of the adrenal steroid synthesis, leading to remarkable side-effects [13]. Later, Fadrozole and Formestane, respectively a non-steroidal and a steroidal inhibitor, were evaluated in BC women, resulting in being safer than Aminoglutethimide, but still inferior to Tamoxifen in terms of benefit [13,15].

Finally, a new generation of AIs, including aromatase non-steroidal, reversible inhibitors, as Anastrozole and Letrozole and a steroidal, irreversible aromatase inhibitor, Exemestane, were developed in the mid-1990s, and radically changed the outcome of post-menopausal hormone-sensitive women with BC [16]. It was demonstrated that these drugs were able to inhibit the synthesis of estrogen that mainly occurs in extragonadal sites (adipose tissue, skin, muscle, bone and central nervous system) in post-menopausal women, and contemporarily affect the enzymatic activity of aromatase in BC cells [17].

Among the third generation AIs, Anastrozole, a benzyl-triazole derivative, is a selective inhibitor of the enzyme, which blocks the electron transfer chain by the cytochrome P450 prosthetic group of the aromatase [17]. It is administered orally, at the recommended dose of 1 mg daily. It reaches the maximum plasma concentration within 2 h, with a half-life of 50 h. It achieves the steady-state concentrations within 7–10 days, and it is metabolized in the liver and excreted in the urine, as unchanged drug (10%) or catabolites (60%) [15]. This drug has been approved for the adjuvant treatment of post-menopausal women with hormone receptor-positive BC just after radical surgery for at least five years, or may be used to continue the adjuvant treatment after 2–3 years of Tamoxifen. Anastrozole alone and in combination with cycline dependent kynase (CDK) inhibitors is similarly recommended for the treatment of metastatic hormone sensitive BC patients [15,18].

Among the other AIs, Letrozole (4,40-bis-benzonitrile) is a selective inhibitor of the enzyme acting with a mechanism of action analogous to that elicited by Anastrozole. It is orally administered at the dosage of 2.5 mg daily, being rapidly and completely absorbed. Its half-life has been calculated in 42 h, however, experimental evidence suggests that it can be much longer in BC patients than healthy subjects. Steady-state concentrations can be achieved in 2–6 weeks of therapy [19]. Similarly to Anastrozole, it is approved for post-surgical adjuvant therapy and for extended adjuvant therapy of hormone sensitive BC patients. Additionally, alone or in combination with inhibitors of CDK, it has been also approved for the treatment of metastatic disease, and in the pre-surgical treatment of locally advanced hormone sensitive BC patients [18,19].

Finally, the last available AIs, Exemestane, is a steroidal antagonist, structurally analogue of androstenedione, the natural substrate of the aromatase. Thus, Exemestane acts as a false substrate, irreversibly linking to the enzyme’s active sites and inducing its permanent inactivation with a consequent accelerated degradation. This drug is orally administered, showing a half-life of 24 h, and achieves its steady-state concentration within 7 days. Alike the other two AIs, Exemestane has been approved for the post-surgical adjuvant treatment of ER-positive BC in post-menopausal women, and as maintenance in those who have already received Tamoxifen for 2–3 years. Additionally, it is also recommended for the second-line treatment of metastatic hormone sensitive BC in post-menopausal women, who show disease progression under Tamoxifen therapy [20].

## 3. Safety and Tolerance Issues of AIs Therapy in BC

The significant clinical benefit and long-lasting survival, which can be achieved with third generation AIs, underline the importance of the compliance to this treatment, that should potentially last for many years. Even though AIs show a positive safety profile, they can induce a variety of adverse events, which often limit the quality of life of these women and therefore, reduce their adherence to the prescribed therapy [21]. The side effects related to these drugs have been partially characterized and greatly diverge for other hormonal treatments, such as SERMs and SERDs [22].

Third generation AIs share side effects which can be observed both in the short-and long-term, and include sexual disorders, menopausal symptoms, impaired cognitive function, cardiovascular events, and musculoskeletal events [23].

Patients treated with AIs often report mild vasomotor symptoms and should consequently adopt significant lifestyle changes, as lowering the temperature of their bedroom, dressing in layers, avoiding triggers, following exercise and diet regimens aimed at a 10% or more loss of body weight, etc. Non-pharmacological treatments, such as cognitive behavioral strategies, hypnosis and acupuncture should also be encouraged, but a sound, systematic and scientifically grounded approach to these problems is not presently available [24]. Conversely, it has been shown that estrogen deprivation induced by AIs exacerbates and increases the frequency of some menopausal nuisances, such as hot flushes, night sweats (with sleep disorders) and fatigue. Anxiety and mild depression occur in about 37% of the cases, and up to 48% when a previous therapy with Tamoxifen had been administered [23]. Some genitourinary disorders may also be associated, that is, urogenital or vulvovaginal atrophy settling the so-called genitourinary syndrome of menopause (GSM), which includes vaginal dryness, itching, irritation, dyspareunia, dysuria and recurrent urinary infections. GSM is a serious concern that may strongly affect quality of life in these patients [25]. In case of moderate to severe menopausal symptoms, selective serotonin reuptake inhibitors, gabapentin or pregabalin and clonidine can be successfully used [24]. The American College of Obstetricians and Gynecologists (ACOG) recommends non-hormonal approaches to manage these symptoms, such as vaginal moisturizers, lubricants and hyaluronic acid gel, as first-line treatment. In patients unresponsive to non-hormonal products, local estrogen therapy can be used, although this strategy should be adopted under the surveillance of the oncologist [26]. In fact, the current evidence does not show any increase in BC recurrence after regular local vaginal estrogens application, but an elevation of serum estradiol levels was observed, suggesting this possibility [25]. A promising treatment for vulvovaginal atrophy could be CO_2_ laser therapy, but its use is presently limited by high costs and availability.

Cardiovascular (CV) events (considered in some instances in the domain of the so-called paraneoplastic syndromes) may be inherently increased in patients with cancer, and include hypertension, venous thrombosis, arrhythmia, cardiac failure, peripheral arterial disease, embolism, myocardial infarction and atrial fibrillation. AIs may increase the risk of CV diseases: the American Society of Clinical Oncology (ASCO) suggests that AIs are associated with ischemic heart disease incidence [27,28], even if an opposite evidence was proposed by a recent systematic review and meta-analysis [29]. Furthermore, a very recent retrospective cohort study based on an outpatient register, covering more than 7 million patients, and so extremely representative of clinical practice, demonstrated no differences in CV diseases between post-menopausal AIs and Tamoxifen users [30].

The possible relationship between CV events and AIs treatment is actually poorly quantified and understood. The most valuable hypothesis points to alterations in lipid metabolism, which follows the fall of protective estrogen levels [23,29]. Unfortunately, a prospective evaluation system, that may allow a reliable estimate of the CV risk for prevention strategies in AIs-treated patients, is not yet available. It was suggested that the administration of dietary supplements, such as folic acid and B6 and B12 vitamins, may reduce CV diseases’ incidence [29,31].

The aromatase enzyme is largely expressed also in the brain tissue, where endogenous 17β2-estradiol is needed for the nervous system functional development and activity, including the cognitive function that may be affected by AIs in the long term [32]. Neurological alterations induced by AIs depend on the period of the life when such drugs are administered. During the perinatal period, or at the onset of sexual maturity, it could disrupt normal organizational/activational programming. In post-menopausal women, AIs therapy has been associated with several negative cognitive side effects (such as difficulty in concentration, forgetfulness and memory deficits, especially in verbal memory) and numbness/tingling of extremities [33,34]. A recent systematic review of related studies suggests that AIs therapy is associated with both short-term and long-term impaired cognitive performances, in comparison with both healthy post-menopausal women, and with BC patients not undergoing AIs. However, there is not uniformity regarding the adopted tests and the affected cognitive domains; furthermore, many revised studies are based on small patient series [34,35]. Additional long-term prospective studies are needed to better elucidate the cognitive consequences of AIs treatment.

## 4. Musculoskeletal Disorders

Adverse events associated with AIs mostly involve the musculoskeletal system and can been classified as (1) metabolic bone disease with a consequent increased risk of fractures; (2) aromatase inhibitors-associated arthralgia syndrome; and (3) autoimmune rheumatic disorders.

The onset of all these adverse events arises after a variable time from the beginning of the treatment. The pathogenetic mechanisms advocated to explain these conditions are mainly related to the estrogen deprivation consequent to a prolonged AIs treatment, however the exact pathophysiology is not fully understood. This review specifically focused on the main clinical features of these three detrimental conditions, the possible underlying mechanisms and consequences, and to provide information concerning either pharmacological or non-pharmacological interventions, to mitigate the entity of these iatrogenic entities.

### 4.1. AIs and Bone Health

#### 4.1.1. Etiopathophysiology of AIs-Induced Bone Loss

The hypo-estrogenic state induced by AIs causes an accelerated bone loss at the trabecular-rich bone sites, and a significant increase in bone resorption. Indeed, estrogen deficiency results in an alteration of the dynamic balance between osteoblast and osteoclast functions. This condition makes T cells prone to secrete tumor necrosis factor (TNF)-α and the receptor activator of nuclear factor-kB ligand (RANKL), which is, in turn, the principle mediator responsible for osteoclast activation. The normal functions of osteoblasts and osteoclasts are in fact sustained through the equilibrium between RANKL and osteoprotegerin (OPG), which is a soluble decoy receptor for RANKL and prevents the binding of RANK to RANKL, inhibiting the osteoclast activity [36].

Multiple genes are involved in the regulation of the bone remodeling process, including those encoding estrogens, vitamin D, insulin-like growth factor (IGF-1), RANKL and OPG. Recently, several single-nucleotide polymorphisms (SNPs) in the above-mentioned genes were found to be associated with AIs-induced bone loss [37]. Particularly, SNPs in the genes encoding estrogen receptors (ESR1 and ESR), in the gene modulating the expression of the enzyme aromatase (CYP19A1) and in CYP11A1 (a gene involved in the steroid pathway) were demonstrated to predict bone density reduction in BC women receiving AIs [38,39,40]. Furthermore, the extensive case-cohort Genome-Wide Association Study (GWAS) identified three SNPs in six estrogen-regulated genes (CTSZ, SLMO2, ATP5E, TRAM2, TRAM14A, MAP4K4), associated with an increase of fracture risk in patients taking AIs [41]. Finally, the genetic variants of the RANK/RANKL/OPG system can be involved in the pathologic bone remodeling observed during AIs therapy. Indeed, the rs7984870 SNP in the RANKL gene resulted in being correlated with an altered RANKL/OPG ratio, with consequent negative impacts on bone health [42].

Bone loss was reported for all the AIs in clinical trials, primarily assessing the efficacy of these drugs in BC, although Exemestane seemed to have a bone sparing effect in preclinical studies, attributed to its androgenic structure [43,44,45,46]. The rate of bone loss during AIs therapy has been reported as two-fold higher than in healthy post-menopausal women [47]. Several clinical trials and a meta-analysis evaluating 30,023 women from 7 RCTs showed a greater fracture risk in patients treated with AIs then those receiving Tamoxifen [48,49,50]. However, the AIs related adverse effects on bone health are less evident in the comparison with placebo, instead of Tamoxifen, probably because the latter is a partial estrogen-agonist and might exert a protective activity [23].

These evidences led to the development of recommendations by the involved scientific societies, defining optimal screening policies and treatment of bone loss during AIs therapy [51,52,53]. Screening for osteoporosis should include detailed patient’s history aimed to identify other risk factors, including familiarity, age, lifestyle, concomitant medications, smoking habit, prior fractures, baseline body mass index (BMI) and measurement of BMD by dual-energy X-ray absorptiometry scan (DXA) at baseline [54,55]. However, some recent reports suggest that both BMD evaluation and low BMI were not accurate predictive factors for fracture risk in AIs-treated patients, since BMD data are associated with vertebral fractures in AI-naïve patients, but not in women receiving AIs [56,57]. This evidence induced to hypothesize that different pathophysiology mechanisms, as those affecting the bone geometry, bone microstructure, and other elements of bone quality, could contribute to the bone fragility in AIs-treated women [58]. In particular, the role of the changes in the bone microarchitecture alterations in AIs-induced osteoporosis was well demonstrated in the study by Marίa et al. [59] Another debated point is the association between adiposity and osteoporosis. In the general population, obesity seems to be associated with a lower risk for osteoporotic fractures, when compared to low values of BMI, possibly due to a protective role of higher estrogen amounts. Conversely, in women undergoing AIs, fat body mass results positively associated with an increased risk for bone fractures, probably due to the loss of estrogen protection, and the oxidative stress and inflammation related to obesity [57].

#### 4.1.2. Management of Bone Health in AIs-Treated Women

A recent algorithm proposed by the European Society of Medical Oncology (ESMO) suggests lifestyle changes, a diet rich in calcium and, if needed, its appropriate supplementation (1000 mg/day), together with vitamin D administration to reach 25(OH) vitamin D serum levels of 30–40 ng/mL, weekly weight-bearing exercise program, limitation in alcohol consumption and smoking cessation, if the T-score (which is the number of standard deviations by which the BMD differs from the mean of an average healthy 30-year-old adult) is more than −2.0, and there are no other risk factors. In these cases, it is suggested to monitor fracture risk and BMD every 1–2 years [52,54,55]. Risk factors able to increase fracture risk in post-menopausal women with BC identified by ESMO are the following: age > 65 years, T-score < 1.5, smoking, BMI < 24, family history of hip fractures, personal history of fragility fracture above age 50, oral glucocorticoid use for >6 months. When the T-score is less than −2.0 and/or more than two, the above risk factors are present, an anti-resorptive treatment should be added to the above-mentioned recommendations [52,54,55]. The therapeutic drug options, presently recommended, are limited to bisphosphonates or Denosumab, considering that the anabolic treatments, as Teriparatide, are not approved in patients with cancer, due to concerns on the possible risk of stimulating tumor progression and recurrence [55]. A large number of trials have been published in recent years, on BC women undergoing AIs and concomitant bisphosphonates, generally demonstrating a significant increase in BMD [60]. The literature data on Denosumab are less available, although, in 2015, the ABCSG-18 random trial showed a significantly lower incidence of fractures and a significant increase of the lumbar spine and femoral neck BMD in BC AIs-treated patients who received Denosumab for 3 years, compared to placebo [61]. Anti-resorptive treatments should be continued, at least until the end of the adjuvant AI-based BC therapy program [50].

### 4.2. AI-Associated Arthralgia (AIA)

Aromatase inhibitor-associated arthralgia is a joint pain disorder occurring in BC patients under AIs treatment. A universally accepted consensus on AIA definition is lacking, and the only available criteria were proposed by Niravath [10] in 2013 (Table 1).

This syndrome consists of symmetrical joint pain, mainly affecting hands, wrists and knees, and sometimes lower back, hips, shoulders and feet. Other common extra-articular manifestations are carpal tunnel syndrome and trigger finger. Furthermore, women experiencing AIA often complain about morning stiffness, myalgia, decreased grip strength, difficulty in sleeping, and fatigue [4,10,62]. The median time of AIA onset after AIs is 1.6 months, although it can range from two weeks to more than 10 months; however, symptoms usually appear within the first two/three months of AIs administration, and tend to peak at the sixth month [63]. The prevalence of AIA in BC post-menopausal women ranges from 20 to 70%; a pooled prevalence of 50% was reported by a recent systematic review [64]. Thus, AIA severely impairs BC patients undergoing AIs, and is one of the leading causes of therapy discontinuation, with non-compliance rates up to 31% at one year; compliant patients at 3 years of treatment can be only 50–68%, according to Beckwé [64], with a possible cancer mortality increase, attributable to intermittent or interrupted AIs administration [65].

#### 4.2.1. Etiopathophysiology of AI-Associated Arthralgia

Very little is known about the pathophysiology of AIA, although several mechanisms have been proposed. Several risk factors, such as a period less than 5 years from menopause, previous menopausal hormone therapy and/or taxanes-based chemotherapy, obesity, pre-existing arthralgia or osteoarthritis (OA) at the beginning of AIs, were highlighted [4,10]. More recently, genetic factors primarily acting in estrogen pathways have been proposed as possibly linked with AIA [66,67,68,69]. Garcia-Giralt et al. [67], in a study on 343 post-menopausal BC women starting AIs therapy, found several SNPs (rs4919686, rs4919683, rs4919687, rs3781287, rs10786712, rs6163) in the CYP17A1 (a gene encoding for the enzymes involved in the biosynthesis of androgens), as correlated with the onset of AIA after 12 months of therapy. Furthermore, the SNP rs1008805 in the CYP19A1 (the encoding gene for aromatase) was shown to be related with joint stiffness and pain in 110 post-menopausal women treated with Anastrozole [70]. These findings were consistent with the study by Park et al. [71], who showed that a haplotype containing this variant was associated with musculoskeletal adverse events in BC patients on Letrozole therapy. Similarly, a Dutch study on 737 women receiving Exemestane reported an association between the incidence of musculoskeletal events during the first year of therapy and the homozygous CYP19A1 rs934635-AA genotype [72]. Moreover, two different studies found correlations between some SNPs of ESR1 gene and AIA syndrome [73,74]. Wang et al. [73] reported that two SNPs (rs2234693 and rs9340799) were associated with the occurrence of musculoskeletal symptoms in 436 post-menopausal women who received Letrozole or Anastrozole; Henry et al. [74] showed that rs9322336 SNP in ESR1 was a predictor of Exemestane discontinuation, because of musculoskeletal side effects. Very recently, a large study on 1049 women treated with AIs found a significant correlation between higher risk of AIA occurrence and SNP rs11648233 in the HSD17B2, the gene encoding for the enzyme responsible for the oxidation of Estradiol (E2) into the weaker Estrone (E1), with consequently lower levels of E2 [69].

Some genetic variants of the vitamin D receptor (VDR) gene were associated with the risk of AIA. Garcia-Giralt et al. [67] in 2013 found rs11568820 SNP in VDR correlated with the onset of arthralgia during the first 12 months of therapy, in AIs-treated women. Subsequently, Niravath et al. [75] analyzed a subset of patients of the “MA.27” study (a phase III adjuvant trial comparing Exemestane vs. Anastrozole), showing that the presence of a Folk-I VDR variant is associated with a significant lower probability of developing AIA. Lintermans et al. [76] demonstrated that OPG rs2073618 was associated with an increased risk of musculoskeletal symptoms and pain, evaluated after 3, 6 and 12 months from the starting of AIs therapy in 254 patients.

Other possible mechanisms responsible for AIA are related to the effects of estrogen deprivation on cartilage and inflammatory system. In fact, it is actually well known that estrogen replacement therapy can improve symptoms like arthralgia and joint pain. However, the role of estrogen on joint tissues is still controversial, although recent evidence supports a chondroprotective effect. Indeed, in a recent study on an ovariectomized rat model of OA, estrogen deficiency correlated with articular cartilage damage and subchondral bone loss, and estrogens administration was shown to reduce the cartilage degeneration [77]. Furthermore, Raloxifene, a SERM, has a documented beneficial chondroprotective effect [78,79]. In particular, this drug was found to induce a significant increase in proteoglycans and a reduction in Metalloproteinases-3 (MMP-3) and nitric oxide (NO) synthesis in human osteoarthritic chondrocytes cultures [78].

Moreover, estrogens have anti-nociceptive properties, mediated by the spinal cord kappa-opioid analgesic system, conceivably due to the evolutionary adaptation process, to help females in tolerating pain during birth labor [4,23]. Unfortunately, there is uncertainty about the threshold level of estrogens at the onset of symptoms and the inter-individual variabilities, that make some subjects more susceptible than others [23]. The role of estrogens in inflammation is also quite controversial. Evidence from the rheumatology literature suggests that high levels of estrogens reduce inflammatory cytokine production, and vice versa; thus, the low estrogen levels of post-menopausal women may induce an increased production of inflammatory cytokines, as Interleukin (IL)-1β and TNF-α [10]. An elevated release of IL-6 was also demonstrated during AIs therapy, and this is probably due to the inhibited activity of aromatase, reducing the expression of this cytokine [80]. From a clinical point of view, the role of the inflammatory process in AIA is supported by an ultrasound (US) and magnetic resonance imaging (MRI) study on 12 women, showing fluid in the sheath surrounding the digital flexor tendons in 5 patients in the US, and the presence of intra-articular fluid in the metacarpal joints, and enhancement and thickening of the digital flexor and extensor tendons of the hands at MRI in all patients [81]. Furthermore, arthralgia was found to be significantly associated with the increase of serum concentrations of inflammatory biomarkers, such as C reactive protein (CRP), eotaxin, monocyte chemoattractant protein (MCP)-1 and vitamin D-binding protein (VDBP), in a cross-sectional study of 203 women taking AIs for early BC [82]. However, the above disclosures and statements on AIA’s pathophysiology are far from conclusive and need further, in-depth studies, in order to identify the therapeutic targets for optimal treatment management. Of course, all the outstanding results of estrogen-based treatment of AIA should be critically considered, due to the harmful potentiality of this approach in BC patients for a possible cancer progression.

#### 4.2.2. Management of AI-Associated Arthralgia in AIs-Treated Women

Several clinical trials have addressed different treatment strategies for AIA, including both classical drugs and procedures, such as vitamin D, steroids, diuretics, Duloxetine, omega fatty acid, Glucosamine and Chondroitin, switching from one AI to another, physical exercise; and even alternative approaches, like herbal remedies, acupuncture, yoga. The results of the pharmacological studies are summarized in Table 2. Low levels of evidence have been achieved in most cases. Presently, there are not standard, uniformly accepted treatments for AIA, and the majority of the proposed algorithms are based on anecdotal reports, or derived from experiences in other pathologies (e.g., arthritis), rather than from specific trials [83].

##### Pharmacological Management of AI-Associated Arthralgia in AIs-Treated Women

Among the pharmacological options in BC women receiving AIs, vitamin D supplementation was investigated, as estrogens are involved in vitamin D activation, in that they potentiate the catalytic activity of 1α-hydroxylase for the conversion of 25(OH)vitamin D into the active form, 1,25(OH)2 vitamin D, as previously mentioned. Furthermore, it should be considered that: estrogens increase the activity of VDR; the majority of patients receiving AIs are lacking vitamin D; and this state can be related to the risk of autoimmune diseases [75]. Again, the results of these studies are conflicting. A first report, addressing the effect of vitamin D supplementation on serum 25(OH)vitamin D levels and AIA symptoms in 60 BC patients undergoing adjuvant Letrozole, dates back to 2010. In this trial, Khan et al. [84] showed that patients with low-baseline vitamin D levels (≤40 ng/mL) who received vitamin D3 at the dose of 50,000 IU weekly for 12 weeks presented a significant improvement of disability (as measured by the Health Assessment Questionnaire II (HAQII)), compared to women who received the standard supplementation with 600 IU/day of vitamin D3 and 1200 mg/day of calcium. Similar findings were reported also by Prieto-Alhambra et al. [85] Furthermore, Rastelli et al. [86] maintained the effectiveness of high dose vitamin D2 supplementation, on the grounds of a small sample study of 60 BC women with AIA onset after starting Anastrozole. Conversely, a double-blind RCT conducted by Shapiro et al. [87] failed to demonstrate a significant beneficial effect of high dose vitamin D3 (4000 IU/day) compared to the standard dose of 600 IU/day, after six months of treatment. A more recent study by Khan et al. [88] also did not achieve the primary end-point, showing no significant difference in preventing new, or worse AIA events, in women starting adjuvant Ais, after either 6-month therapy with oral vitamin D3 at 30,000 IU weekly, or placebo. The most recent RCT by Niravath et al. [89] highlighted the need of further research, examining the role of vitamin D3 supplementation for AIA, especially in order to assess the optimal dosage and the possible individual factors responsible for heterogeneity of patients’ responses to vitamin D. In fact, this trial was prematurely closed for futility after 93 patients enrollment, due to no differences in the onset of AIA, between patients receiving either high dose vitamin D3 (50,000 IU weekly for 12 weeks, followed by 2000 IU daily for other 40 weeks), or standard dose (800 IU daily for 52 weeks). The above studies are methodologically heterogeneous, and do not ground univocal conclusions on vitamin D supplementation in AIA syndrome. In fact, first of all, a standardized definition of AIA is lacking. Second, AIs therapy’s duration differs from one trial to another, as well as the administered vitamin D (D3 or D2). Moreover, some studies focus on the onset of AIA manifestations, other on their worsening and different outcome measures were used, often without an official validation, and sometimes based only on subjective self-assessment.

Other agents employed for the management of AIA syndrome are the omega-3 fatty acids (O3-FAs), based on previous experiences in patients affected by RA, showing effectiveness in reducing joint pain, stiffness, number of swollen joints and use of non-steroidal anti-inflammatory drugs (NSAIDs) [95]. Recently, Shen et al. [91] performed a retrospective exploratory analysis of SWOG S0927, a double-blind RCT, which failed to demonstrate any significant differences in the improvement of AIA arthralgia measured by Brief Pain Inventory—Short Form (BPI-SF) test, between 249 patients treated with O3-FAs vs. placebo, although the placebo effect was greater than expected [90]. In this post-hoc analysis, the Authors also found a significant improvement of BPI after 24 weeks in the obese women receiving O3-FAs, compared to those treated with placebo, a difference absent among non-obese patients. Furthermore, only in obese women, the O3-FAs therapy was associated with a significant improvement of global rate-of-change scores for joint pain and stiffness, with significantly better scores of the Modified Score for the Assessment and Quantification of Chronic Rheumatoid Affection of the Hands (M-SACRAH); and of the Western Ontario and McMaster Universities Osteoarthritis index (WOMAC). It was hypothesized that in obese subjects, the anti-inflammatory effects of O3-FAs could be more evident, considering that these agents might be able to reduce the production of pro-inflammatory cytokines, reactive oxygen species and leukocyte chemotaxis, and that adipose tissue, in turn, is a source of inflammatory mediators [91]. A good tolerability and a potential benefit of O3-FAs on short-term quality of life was also demonstrated in a pilot RCT, on 44 BC women undergoing adjuvant AIs [92].

A large double-blind RCT was recently published, analyzing the effect of Duloxetine, a serotonin-norepinephrine reuptake inhibitor, on pain and quality of life in patients with AIA. In this study, the subjects were treated with oral Duloxetine 30 mg (1 capsule) daily for one week, followed by two capsules daily for 11 weeks, or with matching placebo. At the end of the treatment period, the authors found a greater reduction in average joint pain, measured by BPI, and in joint stiffness in the experimental arm, compared to placebo. Furthermore, a more significant improvement of suitable indexes (WOMAC, M-SACRAH and Functional Assessment of Cancer Therapy-Endocrine Scale Trial Outcome Index (FACT-ES TOI)), was observed in the Duloxetine arm [93]. Subsequently, an exploratory analysis of the same trial demonstrated that obese patients presented a greater analgesic effect from Duloxetine in comparison with non-obese patients, and with obese women receiving placebo [94].

In the last decade, further isolated reports were published, evaluating other potential treatments for AIA. Only a single-arm study on a small patient series addressed the efficacy of corticosteroids in 27 women with AIA, showing a beneficial effect of low-dose prednisolone (25 mg daily) administered for a short period (one week) [96]. However, these results are questionable, due to the short follow-up (two months) and the use of an unvalidated questionnaire [83].

Some studies reported the symptomatic effect of Glucosamine and Chondroitin in different OA localizations [97,101,102]. Recently, a single-arm phase II study on AIA syndrome was published: 53 women received nutritional supplementation with Glucosamine-sulfate (1500 mg/day), plus Chondroitin sulfate (1200 mg/day), for 24 weeks. After 3 months of therapy, 50% of patients experienced 20% or more improvement in pain, stiffness and function (evaluated by WOMAC, M-SACRAH, BPI, FACT-ES TOI), and one third a minimum 20% increase in grip strength. At the closure of the study, an overall 42.6% clinically significant response rate was achieved, as defined by the Outcome Measures in Rheumatology Clinical Trials and Osteoarthritis Research Society International (OMERACT-OARSI) criteria [97]. These results are limited by the uncontrolled design of the trial; furthermore, the agents under evaluation were administered as dietary supplements, that is, not according to formal medical prescription formulations of Glucosamine sulfate and Chondroitin sulfate [103].

Another promising option in AIA’s symptoms management is vitamin B12. Administered orally at the dosage of 2500 mcg daily for 90 days, as a dietary supplementation, it was shown to reduce pain, as evaluated by BPI, and improve quality of life, as determined by FACT-ES TOI in a single arm phase II study [98]. Obviously, large controlled trials, based on formal medical prescription schedules, are necessary to validate these suggestive results.

A retrospective analysis included 288 patients chronically assuming diuretics for hearth diseases or hypertension, and receiving AIs therapy for BC: it has been reported that they were less likely to develop musculoskeletal symptoms [104]. On these grounds, the potential effects of diuretics in AIA syndrome were tested in a prospective phase II trial: fifty post-menopausal AIA patients received an oral combination of Frusemide 20 mg and Spironolactone 50 mg every other day for 4 weeks. A significant reduction of pain, stiffness and functional disability was detected by WOMAC and quick Disabilities of the Arm, Shoulder and Hand (DASH) score [99].

Bisphosphonates were investigated as possibly active drugs against AIA, based on their already known efficacy in decreasing bone loss and improving BMD. A prospective phase II, single arm trial, was aimed at evaluating the treatment with zoledronic acid in reducing the incidence of AIA. Fifty-nine post-menopausal BC women received zoledronic acid (4 mg i.v.), 1–2 weeks before Letrozole, then after 6 months. A significantly lower incidence of AIA at a 1-year follow-up was shown in patients receiving zoledronic acid, compared with historical controls from the Exemestane and Letrozole Pharmacogenetics (ELPh) trial [100].

The possibility of managing AIA through a switch from one AI to another, with potentially milder side effects, was also investigated. Briot et al. [105] administered to 179 patients affected by AIA, Letrozole for 6 months, after Anastrozole discontinuation, and one-month washout, in a single-arm prospective study. At the end of the 6-month study, the majority of the patients (71.5%) were still taking Letrozole, and a significant decrease in BPI (and also significant improvement in the physical and mental components of SF-12) was observed, in comparison to the time of Anastrozole discontinuation.

##### Non-Pharmacological Management of AI-Associated Arthralgia in AIs-Treated Women

The possible role of exercise was maintained, among the non-pharmacological options for AIA syndrome. Current guidelines recommend exercise as a part of the routine lifestyle of women with BC: the American College of Sports Medicine suggests to cancer survivors aerobic and resistance training for about 30 min, for three sessions per week [106,107]. Furthermore, several RCTs and systematic reviews dealt with different techniques, as cardiovascular and resistance exercise, yoga, tai-chi, swimming, walking and pilates, emphasizing the beneficial effects on AIA, that is, pain, stiffness, grip strength and quality of life improvements, particularly when professional guidance is achieved [108,109,110,111,112,113,114,115]. However, a very recent Cochrane review, including seven RCTs for a total of 400 enrolled patients, provided no clear scientific evidence in favor of exercise in BC women with AIA. Criticisms have been raised regarding methodological heterogeneity in many aspects of the considered studies, such as type, frequency, intensity and duration of practices, as well as lacking comparisons with suitable control arms and supervision validations [116]. Finally, among the treatments of AIA based on non-conventional medicine, some interest was dedicated to acupuncture, which is maintained to enhance the endogenous production of opioid peptides and increasing blood flow [117]. In the last decade, several RCTs were published in this regard, rarely showing acupuncture more effective than sham [118,119]; in fact, statistical evaluations did not show any significant differences [120,121,122].

### 4.3. Rheumatic Autoimmune Diseases

A growing literature evidence of a close link between AIs therapy for BC and rheumatic autoimmune diseases has recently emerged, and the main characteristics of the related studies are summarized in Table 3. However, many aspects, such as incidence and prevalence, time of onset and probability of remission, are not yet completely quantified.

#### 4.3.1. Literature Data on the Association between AIs and Autoimmune Rheumatic Diseases

A small deal of literature dating back to the 1990s of the last century described, as case reports, the occurrences of RA in patients undergoing Tamoxifen [133,134]. Subsequently, a well detailed case of association between AIs therapy and RA was reported in 2007: a patient affected by advanced BC treated with Exemestane complained about joint stiffness and pain in hips, shoulders, knees, wrists and hands, just after a few days of treatment. After four weeks, a typical symmetric and active arthritis appeared, with the involvement of wrists, metacarpo-phalangeal (MCF) and proximal inter-phalangeal (PIP) joints, and did not relieve after Exemestane discontinuation. The laboratory workup showed erythrocyte sedimentation rate (ESR) and CRP increase, and radiographs and MRI imaging of the hands documented typical erosions, allowing a diagnosis of RA. Methotrexate (15 mg/week) was started, and a significant improvement of the Disease Activity Score (DAS), including 28-joint count, was observed after four months [123]. Another single case of RA associated with Anastrozole was reported in 2011, occurring after one year of treatment: a 56-year-old BC patient reported widespread arthralgias that evolved three years later in an active RA, documented by increase of rheumatoid factor (RF) and anti-cyclic citrullinated protein (CCP) antibodies [124]. According to the American College of Rheumatology (ACR) criteria [135], RA in a high activity phase was diagnosed and therapy was started with Methotrexate (15 mg/week), Methylprednisolone (16 mg/day), bisphosphonate and vitamin D, with a significant clinical improvement after three months [124]. In the same year, Bertolini et al. [125] published a case series of 3 BC women who developed RA during AIs therapy (Anastrozole in 1, and Letrozole, followed by Exemestane in the other 2 patients). The symptoms (mainly hands arthralgias and morning stiffness) occurred after a few weeks from the AIs initiation, while the diagnosis of RA was made after a mean period of 33 months. All patients showed immunological markers of RA (anti-CCP and/or RF) and hand radiographs showed typical erosions in one patient. All three women responded satisfactorily to conventional medications for RA, such as Hydroxychloroquine, Sulfasalazine and Prednisone. Furthermore, in a long-standing RA patient, Letrozole seemed to be responsible for the occurrence of accelerated cutaneous nodulosis, characterized by the presence of multiple small subcutaneous nodules on the fingers of both hands. This particular RA manifestation was reported after 16 months of AI therapy. After drug discontinuation, a slow decrease in the size and tenderness of the nodules was observed [126].

Other than RA, other types of definite arthritis were found to be associated with AIs treatment. In fact, in a report by Scarpa et al. [127], 10 out of 18 AIs-treated post-menopausal women referred for rheumatological evaluation due to joint complaints, were diagnosed, as affected by undifferentiated SpA, two with oligoarthritis and the other six with simple arthralgia. Almost all patients (16/18) were treated with NSAIDs or with corticosteroids; in three non-responder cases, Methotrexate was added, while AIs discontinuation was needed for two patients who subsequently experienced a spontaneous resolution of symptoms.

Furthermore, in recent decades, many other reports have dealt with the subject of the association between AIs and other autoimmune diseases, in small BC patients’ series with various methodologies and endpoints. Laroche et al. [128] reported their findings out of 24 patients, applying for rheumatologic consultation during AIs therapy for pain greater than 5/10 on a visual analog scale. Ten patients were affected by sicca syndrome of the eyes and mouth probably due, or due for sure, to SjS in nine and one cases, respectively, according to the San Diego criteria [136]. In the remaining cases, OA, shoulder tendinitis or paraneoplastic aponeurositis were identified as the cause of pain in five patients. An abnormal autoantibody positivity was present in nine cases (antinuclear antibody titer > 1/160) [128]. An association between AIs and SjS (according to the 2002 European criteria definition [137]) was subsequently reported in 3 BC patients [129]. All of them complained about diffuse arthralgia and eyes and mouth dryness, with symptoms’ onset from three to five months after the start of AIs. The salivary gland biopsies revealed a Chisholm and Mason stage 4 SjS. Another occurrence of SjS complicated with neuropathy of both legs, after Anastrozole therapy for BC, was described by Yasar-Bilge et al. [130]. The authors maintained that other possible causes of neuropathy (chemotherapy side effects, paraneoplastic manifestations, cryoglobulinemia-related vasculitis) could be excluded, thus they hypothesized a causal relationship between AIs and SjS.

A case of SS in a very early phase was observed by Pokhai et al. [131] during AIs therapy. After two years of treatment with Letrozole, the patient reported hand joint pain and stiffness, and two years later, she developed bilateral indurations of the dorsum of the hands and distal vasospastic phenomena exacerbated by exposure to low temperature, consistent with the Raynaud phenomenon. Her laboratory analysis showed a high titer of anti-nuclear antibody (1:1280) with a centromeric pattern, and positive anti-centromere-B antibodies. The diagnosis of SS was made, and Letrozole was switched to Exemestane, with relief of articular pain. After the end of the 5-year AI treatment, she experienced less vasospasm of the fingers, improvement of the skin induration, resolution of the periungual erythema, and increased movements of the fingers.

In 2016, Mascella et al. [132] reported a case of ASAS after treatment with AIs, in a BC patient with a previous diagnosis of RA, who developed a severe bilateral interstitial pneumonia and necrotizing myopathy, associated with a creatine kinase increase and positivity of anti-Jo1 and anti-Ro52 antibodies, after 3 months of therapy with Letrozole. After the withdrawal of hormone therapy and introduction of high-dose steroids, in addition with Azathioprine, the myositis and the interstitial lung disease significantly improved, but a re-exacerbation was reported, following the introduction of a second AIs agent (Anastrozole). The time correlation between AIs administration and the occurrence of the clinical manifestations, and the rapid re-occurrence, strongly supported an etiological relationship.

Recently, we described the case of a 56-year-old woman with primary APS (defined according to the 2006 updated criteria [138]), occurring after six months of Anastrozole treatment for BC [11]. The diagnosis was made after recurrent episodes of cerebral ischemia and the detection of a triple positivity for anti-phospholipid antibodies (aPL) (lupus anti-coagulant plus anti-cardiolipin antibodies plus anti-β2 glycoprotein 1 antibodies). We initially administered Enoxaparin, and subsequently oral Warfarin, to reach the recommended INR value of 3–4. As soon as INR values were achieved, we added oral Hydroxychloroquine, at the dosage of 200 mg twice a day and intravenous human immunoglobulins (400 mg/kg/day for five consecutive days), these latter followed as maintenance by monthly cycles of low-dose (400 mg/kg/day in a single infusion), according to a previous dose protocol experienced in SLE, discoid lupus erythematosus (DLE), primary and secondary APS [139,140,141]. Anastrozole was not discontinued until the end of the recommended 5-year schedule. Clinical conditions were stable after a 6-year follow-up, with no subsequent ischemic or thrombotic event.

Furthermore, some reports of autoimmune hepatitis and dermatologic autoimmune conditions (erythema nodosum, skin vasculitis, and subacute cutaneous lupus erythematosus), related to AIs therapy for BC, may be of some interest [142,143,144,145].

The recent report by Tarhan et al. [146] deserves a particular consideration. This is a study on the distribution of rheumatic diseases in BC patients referring for musculoskeletal complaints to the Rheumatology Outpatients Clinics of two hospitals in Turkey from 2008 to 2018, excluding those with a previous diagnosis of a definite rheumatic disorder and/or with bone metastasis. Out of the 128 patients included, nearly one third (32.03%) developed autoimmune rheumatic diseases, mainly RA, and in lesser percentages, SjS, psoriatic arthritis, SS, gout arthritis, Behçet’s syndrome, SLE, ankylosing spondylitis and non-radiographic axial SpA. The ten patients who presented RA had received Tamoxifen, and not AIs for BC. This observation may raise the question of a possible non-specificity of AIs-induced rheumatologic-autoimmune disease.

#### 4.3.2. Literature Data on the Incidence of Rheumatic Diseases during BC Hormone Therapy

The incidence of rheumatic diseases during various anti-estrogens therapies for BC was also explored by some formal retrospective analyses during the last decade (Table 4).

Chen et al. [147] evaluated the risk of SLE or RA out of 238,880 BC patients undergoing SERM or AIs therapy in USA. They could show an increased risk of RA for patients exposed to both these categories of drugs, compared to the general population, more evident for a long-time exposure (>12 months) to SERMs. Only the patients receiving SERMs resulted in being at higher risk of SLE.

Caprioli et al. [148] conducted a retrospective cohort study out of 7533 BC patients submitted to mastectomy and adjuvant treatment with Tamoxifen or AIs, and included in the healthcare database of Lombardy (Italy) from 2004 to 2013. Considering the 26,105.9 person-year, a total of 113 new cases of RA occurred (26 in the Tamoxifen sub-group and 87 in the AIs sub-group), corresponding to a crude incident rate (IR) of 4.33 per 1000 person-years (95% CI 3.57 to 5.20). Using Tamoxifen as a reference category, AIs therapy was associated with an increased risk of RA (adjusted hazard ratio (HR) 1.62 (95%1.03–2.56)), mainly in patients receiving Anastrozole, even after adjusting for age and stage of neoplasia (adjusted HR 1.75 (95%1.07–2.86)).

Chien et al. [149] performed an analysis, based on the Taiwan national health insurance research database, on BC patients treated with Tamoxifen or AIs. AI therapy was associated with significantly higher one-year cumulative incidence for any kind of arthritis and carpal tunnel syndrome, compared to Tamoxifen. However, these authors included in the “any arthritis” category OA, RA and others arthritis, without any distinction.

Interestingly, Wadstrom et al. [150] recently analyzed the risk of incident BC in women with RA diagnosed from 2006 to 2016, and the risk of RA in women with a history of BC, using nationwide Swedish registers. The authors found a reduced risk of incident BC in RA women compared to the general population; similarly, a decreased risk of RA in women with a history of BC was observed. Furthermore, treatment of BC with Tamoxifen and AIs did not result in being a risk factor for the onset of RA.

#### 4.3.3. Etiopathophysiology of AIs-Induced Rheumatic Autoimmune Diseases

**Figure 1:**
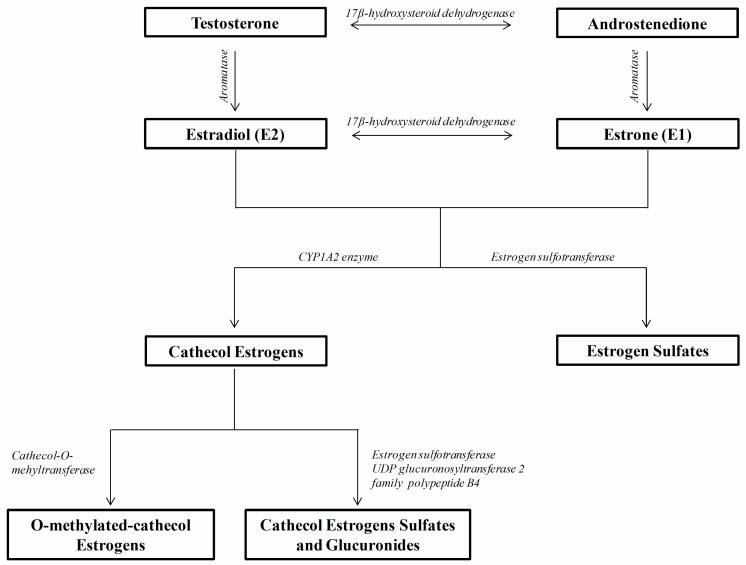
Biosynthesis and metabolism of estrogens.

The pathogenetic mechanisms responsible for the occurrence of rheumatic autoimmune diseases during AIs therapy are not yet satisfactorily known, on the whole. As previously outlined, aromatase is the enzyme responsible for the conversion of androgens to estrogens: in particular, it converts androstenedione to estrone and testosterone to estradiol (Figure 1). Thus, aromatase blockade results in estrogen depletion which has been well demonstrated as a consequence of AIs therapy, while only few studies considered the change in androgens concentrations during the course of the treatment, with conflicting results [151,152,153].

In general, the role of estrogens is certainly pivotal in autoimmune diseases, but it is also very complex. The prevalence of autoimmune rheumatic conditions in female patients, especially at reproductive ages, and the influence of the menstrual cycle, pregnancy and menopause on the course of these pathologies demonstrate a central mechanism of estrogens in the pathophysiology of autoimmunity [154,155]. However, this composite framework is complicated by the evidence that estrogens may exert opposite effects on immune system. In fact, the estrogens’ activities seem to depend on their different concentrations, the phase of the disease in which they act, their ability to generate various types of active metabolites, and the efficiency of the functional estrogen receptors, affected in turn by the microenvironment and by the kind of disease [154,156]. These variables make it difficult to understand the role of estrogens in autoimmune diseases, and even more hard to know how the aromatase inhibition may influence the pathophysiology of these disorders. A challenging question is whether the hypoestrogenic state induced by AIs is a facilitating factor for the development of autoimmune diseases, or if other mechanisms could be taken into major consideration.

Presently, most scientific evidence derives mainly from preclinical evaluations. A study on an experimental rat model of RA reported a significant association between the estrogen deprivation induced by Anastrozole and the enhancement of RA severity. In particular, the production of pro-inflammatory cytokines, such as Interferon (INF)-γ and IL-12 (Th1-related cytokines), was significantly stimulated, after Anastrozole administration, while the anti-inflammatory cytokines, such as IL-4 and IL-10 (Th2-related cytokines), were inhibited. Thus, Anastrozole was shown to alter Th1/Th2 balance in favor of Th1, that is considered crucial in the pathogenesis of Th1-mediated immune diseases, as RA. Moreover, Anastrozole down-regulated the CD4+CD25+Foxp3+ Treg population, which plays an important role in the control of immune system, preventing autoreactive responses [157]. The effect of AIs on the modulation of Treg cells has been demonstrated also for Letrozole in a human in vivo study, where Treg expression in tumor samples of BC women treated for 6 months with Letrozole resulted in being significantly decreased; additionally, it was also shown that this T cell subset decline was directly correlated with the response to the treatment, suggesting that AIs can indeed also exert additional immunomediated anti-tumor effects [158]. Treg down-regulation could also have significant anti-cancer implications to be considered in the development of news coming oncologic immunotherapy trials with programmed cell death receptor-1 (PD-1)/PD-1 ligand-1 (PDL1) immune checkpoint monoclonal antibodies inhibitors. The latter rescue tumor-infiltrating cytotoxic-T-lymphocytes (CTLs), inactivated throughout the PD-1 pathway due to interaction with PDL-1/2 molecules expressed on inflammatory and cancer cells [159].

Further studies on animal models provided some evidence that the aromatase blockade is responsible for the onset of pathological autoimmune alterations, similar to those observed on SjS in humans. In this regard, Shim et al. [160] demonstrated that female Aromatase gene knockout (ArKO) mice spontaneously developed a destructive infiltration of B lymphocytes in the salivary glands, resembling human SjS exocrinopathy. This finding led us to hypothesize that an excess in B cell survival (a key event in the pathogenesis of SjS-related disorders) may be caused by the up-regulation of the estrogen-regulated anti-apoptotic protein B cell lymphoma (Bcl)-2 [161]. In addition, ArKO mice presented impaired renal function with proteinuria and proteolytic fragments of α-fodrin in the salivary glands (as typically found in human SjS), as well as anti- α-fodrin antibodies in the serum. Furthermore, a diet containing phytoestrogens seemed to prevent the development of these autoimmune alterations, confirming the strong implications of estrogens in immune-regulation [160]. Similar results were recently achieved by another study, showing SjS-like inflammatory lesions in lacrimal and salivary glands of ArKO mice and increased autoantibody production. Moreover, the autoimmune lesions in these tissues were exacerbated by the intra-peritoneal injection of Exemestane. The authors also found a significant amount of white adipose tissue in ArKO mice compared to their wild-type counterparts used as controls, as well as accumulating macrophages and increased MCP-1 expression in the adipose tissue, with consequent enhanced release of pro-inflammatory cytokines, as IL-1β, IL-6, IFN-γ and TNF-α. Similarly, the salivary glands showed an increase of adiposity and of the MCP-1 expression levels. These authors suggested that the production of the above mentioned pro-inflammatory cytokines and mediators by macrophages can disrupt the local immune tolerance in the salivary gland, and trigger the onset of SjS. However, the molecular mechanism which links the aromatase enzyme with this autoimmune disease is not yet fully understood [162]. The results of this study suggest an immune-modulating effect for the adipose tissue, probably due to its ability to produce pro-inflammatory adipocytokines, such as leptin, adiponectin, visfatin, IL-6, IL-8, etc. that, in turn, may account for the complex relationship between obesity and autoimmune diseases [163,164,165]. Few data are available about the influence of AIs therapy in patients with BC on body composition [166,167]. A recent, well designed study showed a greater percentage of body fat and higher plasma leptin concentration in post-menopausal women taking AIs for BC, in comparison to control subjects with no history of BC [168]. This increase of adipose tissue and of circulating leptin could contribute to the induction of autoimmune diseases by these drugs.

As suggested also by Melillo et al. [169], in a recent review on the possible molecular mechanisms responsible for the onset of autoimmunity during AIs therapy, other biological pathways may involve IL-17. In a genome-wide association study, a SNP signal on chromosome 14 that mapped near the 3’ end of the T-cell leukemia 1A (TCL1A) gene, was identified as being associated with musculoskeletal pain in women in adjuvant AIs therapy for BC. This SNP resulted in an increased expression of TCL1A which, in turn, up-regulated IL-17 receptor (IL-17RA) expression and down-regulated the expression of IL-17, IL-12, IL-12RB2 and IL-1R2 [170]. IL-17 plays a major role in inflammatory arthritis, particularly psoriatic arthritis and spondyloarthritis. In this context, IL-17 acts as a key amplifier of the inflammatory response, as it initiates the synthesis of several other inflammatory mediators, such as granulocyte-macrophage colony stimulating factor, Prostaglandin (PG)-E2 and IL-8, which, in turn, increase the inflammatory cascade [171]. The estradiol-dependent regulation of this cytokine and of its receptor expression, mediated by TCL1A, might help to explain the association of TCL1A with musculoskeletal symptoms, within the range of SpA described in patients treated with AIs [127,169]. Other pro-inflammatory cytokines, such as TNF-α and IL-1β, are known for their central role in the pathogenesis of autoimmune diseases, and previous evidence showed that estrogens modulated their release, controlling the expression of CD16 receptor on monocytes and macrophages [172]. However, a significant difference in the serum concentrations of these inflammatory markers between AIs-treated women reporting rheumatological complaints and those receiving the same therapy, but without any rheumatic symptoms, is not yet demonstrated [173].

Another interesting point regards the effects of AIs and vitamin D, well known as an important regulator of immune function and inflammatory processes [174]. Low vitamin D levels have been showed in several autoimmune diseases, and beneficial effects were often reported after high dose vitamin D supplementation in rheumatological and non-rheumatological disorders [175,176,177]. Vitamin D regulates both innate and adaptive immunity, and the conversion in its active form 1,25(OH)2 vitamin D can occur in immune cells, such as dendritic cells, macrophages, T and B cells [178]. Moreover, 1,25(OH)2 vitamin D stimulates the secretion of IL-10 (an anti-inflammatory cytokine) and reduces the production of pro-inflammatory cytokines, such as IL-1, IL-6, IL-8, IL-12, TNF-α and INF-γ [179].

The low estrogen levels induced by AIs drugs decrease the availability of 1,25(OH)2 vitamin D, because sexual hormones increase the activity of 1α-hydroxylase and potentiate the activation of VDR [75]. Furthermore, different authors demonstrated insufficient levels of vitamin D in about 75–90% of women receiving AIs therapy [180,181]. Thus, vitamin D deficiency can represent another risk factor for the development of autoimmune disorders during AIs therapy.

From a different point of view, it could be considered the possible role of an inherent relationship between BC and autoimmune diseases, such as SjS and SLE, attributable to the Sjogren syndrome-associated autoantigen (SSA), also known as Ro52 and TRIM 21. SSA belongs to the TRIM (Tripartite motif) family of proteins and it exerts a E3 ubiquitin ligase activity, mediating ubiquitination of several members of the interferon regulatory factor (IRF) family. Its pathologic role in SjS or SLE was demonstrated by a study with TRIM21 null mice, developing systemic autoimmune diseases. This molecule is an important regulator of immune homeostasis, particularly, it negative modulates the pro-inflammatory cytokines. Thus, a loss of function of this key regulatory protein can result in exaggerated tissue inflammation and systemic autoimmunity [182]. Growing evidence has shown that TRIM21 is also involved in the progression of human cancers: low TRIM21 expression was correlated with poor overall and disease-free survival in two independent cohorts, accounting for 1219 BC patients. The multivariate Cox regression analysis revealed also that TRIM21 is as an independent factor for overall survival, and its over-expression inhibited cell proliferation and tumor growth, enhanced instead by TRIM21 depletion, leading to the hypothesis that TRIM21 may have a role as a tumor suppressor in BC, with potential prognostic and therapeutic biomarker value [183].

In summary, different hypotheses, probably cross-linked to each other, were suggested to explain the relationship between AIs and autoimmune disorders. The most explored one supports the role of estrogens in autoimmune diseases, with the estrogens’ deprivation representing a potential triggering factor for autoimmunity. Other theories suggest that the influence of adipose tissue, of the vitamin D deficiency, and further determinants, can contribute to the development of immune response during AIs therapy.

Interesting findings may derive, in the future, from the investigation of another pathophysiology domain, represented by the interaction among major histocompatibility complex (MHC), autoimmune diseases and AIs therapy. In fact, the strong association between the MHC genomic region and autoimmune diseases has been established for over 50 years, as well as the role of human leucocyte antigen (HLA) (located within the MCH) in the development and progression of cancer [184,185]. However, there are presently no studies investigating specific HLA genes, as susceptibility factors for autoimmune disorders in BC women treated with AIs.

## 5. Conclusive Remarks

Aromatase inhibitors therapy has radically changed the prognosis of hormone receptor positive BC in post-menopausal women in the last thirty years, and is still a mainstay of therapy in the adjuvant and advanced stage settings [186]. The anti-cancer effect of AIs mainly consists of the inhibition of aromatase enzyme, consequently inducing estrogen deprivation, which in turn is responsible for the inhibition of cell proliferation, through the cell cycle arrest in G0/G1 phase, and enhanced apoptosis in BC cells. Indeed, in mammary glands, estrogens are known to act as mitogens, stimulating cell proliferation, by modifying the expression of hormone-responsive genes involved in cell cycle and/or programmed cell death, and through the activation of ER. In addition, these steroidal hormones inhibit apoptosis by ER-mediated mechanisms and by the regulation of the expression of several apoptotic factors, including Bcl-2 [2].

However, this effective treatment comes at the cost of some detrimental side effects, which significantly impact the patients’ adherence to care. The main adverse events which may lead to premature AIs therapy discontinuation involve the musculoskeletal system, in the form of bone loss, AIA syndrome and autoimmune rheumatic diseases. Some of these conditions, as AIs-induced osteoporosis, are the direct consequence of the hypo-estrogenic state derived from the aromatase blockade, and were extensively investigated in the last decade. Several recommendations were drawn by the representative scientific societies, and are presently available for the screening and the management of bone loss during AIs treatment.

The other non-autoimmune and autoimmune musculoskeletal side effects, occurring during AIs administration, are less foreseeable and unsatisfactorily explored, in our opinion. This narrative review deals, among them, with the AIA syndrome, which strongly compromises the completion of AIs therapy, with a possible, increased mortality for cancer. We are aware that this is a high prevalent condition, but a precise knowledge of this disorder is still impaired by the lack of clear and universally accepted definitions of the pathogenetic mechanisms, which should be the ground for both the diagnostic workup, and the guidelines for the treatment. However, several theories have been proposed in this regard, in general confirmed by limited methodological evidences. In fact, their clinical background is derived mostly from limited series or anecdotal reports, whereas many interpretations are borrowed from other fields, such as arthritis. A mounting interest in pharmacogenetic studies aimed at identifying possible genomic markers, which may predict the occurrence of AIA syndrome, and help clinicians in selecting the patients who better benefit from the full course of AIs therapy, recently arose. Unfortunately, the pharmacological and non-pharmacological management of AIA syndrome is not yet fully assessed.

Furthermore, it is of the utmost interest, both from the preclinical and clinical sides, the association of AIs therapy with autoimmune diseases, mainly RA and SjS. The related literature has been recently expanded by a growing number of publications on pathogenetic mechanisms, which may link autoimmunity with AIs treatment, with not always coherent and conclusive results. In fact, from these studies, considered as whole, it emerges that the increased autoimmunity risk is probably not related only to the hypo-estrogenic condition induced by AIs. The main factors limiting the clinical reliability of this scientific domain are, also in this case, the small number of patients included in the evaluated series, and the prevalent retrospective nature of the studies, often based on healthcare registers, thus allowing incomplete information. On the other hand, preclinical investigation covers only limited domains of the complex pathophysiology landscape of the rheumatologic iatrogenicity of AIs. Furthermore, the management of these disorders is strongly conditioned by the impossibility of using all the effective treatment resources presently available in rheumatology, as the so-called “biologics” drugs, including anti-TNFα, anti-IL1, anti-IL6, anti-CD20, T-cell activator inhibitor, anti-IL17 and anti-IL12/23. The term “biologics”, in general, refers to a variety of treatments of natural origin (vaccines, blood components, gene therapy, and recombinant proteins), but it became typically used to refer to a subgroup of large, complex molecules used for targeted therapy, including monoclonal antibodies and receptor fusion proteins [187]. The safety of these drugs in oncologic patients is not fully known, and it was hypothesized that these agents could reduce tumor surveillance [188].

Considering all these data on the AIs iatrogenicity as a whole, and in particular the high incidence of bone metabolic disease and AIA syndrome and the difficulty of treating rheumatic autoimmune conditions, we think that the scientific community should become aware of these concerns. Furthermore, we believe that it is very important for both oncologists and rheumatologists to establish a sound and effective cooperation, in order to overcome a limit impairing the successful use of this kind of hormone therapy in a significant proportion of BC patients.

However, the advent of Fulvestrant, with indications partially overlapping those of AIs (first- and second line hormonal therapy in advanced and metastatic BC), may give rise for the question if comparable, or even improved therapeutic outcomes are achievable by the former, with less toxicity, although there are no reliable disclosures on non-autoimmune and autoimmune rheumatological musculoskeletal adverse effects related to SERDs [189,190].

The main limitation of this review lies in its narrative nature with all the limitations inherent to a non-rigorous systematic review. In particular, this paper did not identify the quality and the strength of the mentioned studies, and has not been built on a robust methodology structure. Furthermore, the completeness of the information presented may be influenced by the inclusion of articles only written in English, and by a search not considering all existing databases, such as EMBASE. Then, since data extraction has been done only by two researches of the same expertise area (rheumatology), errors and biases cannot be excluded. Besides, the current review aimed to provide a general overview on the musculoskeletal disorders associated with AIs therapy, and not to draw a conclusive remark, considering the heterogeneity of the analyzed studies and the variety of the explored topics.

In conclusion, the ultimate goal of the present article would be to stimulate the filling of the relative void of suitable scientific information on this field, through new, suitable methods of scientific investigation. Given the great complexity of this field of investigation, big-data prospective collections from the “real world”, with the uniformity of “ontologies”, including bone side effects, sign and symptoms of the AIA syndrome and clinical and laboratory assessment for autoimmune diseases, at baseline and at regular intervals during the therapy, should be advisable through the cooperation of the oncologic and rheumatologic scientific communities. In fact, such advanced methods of analysis, based on artificial intelligence and machine learning, have been recently suggested for rheumatic and musculoskeletal disorders [191]. We think that the biomolecular studies for identifying biomarkers predicting high risk for bone loss, AIA syndrome and autoimmune diseases could be promoted in such a translational context. This may also provide sound elements for patients’ information, besides the main target of improving their quality of life and survival probability.

## Figures and Tables

**Table 1:** Definition of Aromatase Inhibitors-induced Arthralgia (AIA) according to Niravatah et al. [10]

**Major criteria**
Currently taking AIs therapy
Joint pain which has developed or worsened since starting AIs therapy
Joint pain improves or resolves within 2 weeks of stopping AIs therapy
Joint pain returns upon resuming AIs
**Minor criteria**
Symmetrical joint pains
Pain in hands and/or wrists
Carpal tunnel syndrome
Decreased grip strength
Morning stiffness
Improvement in joint discomfort with use or exercise

Abbreviations: AIs: Aromatase Inhibitors.

**Table 2:** Characteristics of the studies analyzing different pharmacological interventions for Aromatase Inhibitors-associated arthralgia (AIA) syndrome.

Authors	Study Design	Pts (no)	AIs	Interval Time between AIs Starting and the Studied Treatment	Interventions Arms	Study Duration	Follow-Up Duration	Adherence to the Whole Protocol	Significant Outcomes
Khan et al. [84] 2010	Prospective study	60	LTZ	4 weeks	Arm 1 (47 women with 25OHD levels ≤40 ng/mL): 50,000 IU of oral VitD3/week Arm 2 (13 women with a 25OHD level > 40 ng/mL): calcium 1200 mg/day and VitD3 600 IU/day	12 weeks	12 weeks	85%	Higher (*p* = 0.059) improvement of HAQ-II in arm 1 vs arm 2 at the end of the therapy. No significant change of BFI, MEN-QOL and subjective joint pain between the two groups
Prieto-Alhambra et al. [85] 2011	Prospective not controlled study	260	N.R.	Started together	Arm 1: oral 16,000 IU VitD3 every 2 weeks, in addition to oral calcium (1 g) and VitD3 (800 IU) daily	3 months	3 months	97.6%	VAS joint pain was significantly (*p* = 0.02) attenuated in patients reaching concentrations of 25OHD of ≥40 ng/mL, with a lower risk of incident arthralgia
Rastelli et al. [86] 2011	RCT	60	ANA	8 weeks	Stratum A (women with 25OHD levels 20–29 ng/mL): oral 50,000 IU VitD2 (Arm 1) or oral placebo (Arm 2) weekly for 8 weeks, then monthly Stratum B (women with 25OHD levels 10–19 ng/mL): oral 50,000 IU VitD2 weekly (Arm 1) or oral placebo (Arm 2) for 16 weeks and then monthly	6 months	6 months	78%	Pain severity, as measured by FIQ and BPI-SF significantly decreased in patients treated with VitD vs placebo after 2 months, but at 6 months follow-up there were no significant differences
Shapiro et al. [87] 2016	RCT	116	LTZ: 55 pts ANA: 47 pts EXE: 11 pts	Mean ± SD: 19.9 ± 17 months	Arm 1 (56): oral 600 IU VitD3 plus 1000 mg calcium carbonate daily Arm 2 (57): oral 4000 IU VitD3 plus 1000 mg calcium carbonate daily	6 months	6 months	95%	No significant differences between the groups in BCPT-MS scale, PROMIS score, HGST, AUSCAN and WOMAC at 6 months
Khan et al. [88] 2017	RCT	160	LTZ	Started together	Arm 1 (80 pts): oral 30,000 IU VitD3 weekly, in addition to 1200 mg of calcium and 600 IU of VitD3 daily Arm 2 (80 pts): oral placebo weekly, in addition to 1200 mg of calcium and 600 IU of VitD3 daily	24 weeks	24 weeks	91%	30,000 IU VitD3 weekly failed to show a benefit in preventing new or worsening AIA based on the protocol defined primary endpoints (HAQ-II, CPIS, LTZ discontinuation)
Niravath et al. [89] 2019	RCT	93	N.R.	Started together	Arm 1 (46 pts): oral 50,000 IU VitD3 weekly for 12 weeks, followed by 2,000 IU daily for 40 weeks Arm 2 (47 pts): oral 800 IU VitD3 daily for 52 weeks	52 weeks	52 weeks	89%	12 weeks after randomization, 57% from arm 2 and 54% from arm 1 developed AIA (defined as an increase of HAQ-II ≥ 0.2 and/or an increase of VAS pain ≥ 0.3) and the study was terminated early for futility
Hershman et al. [90] 2015	RCT	249	ANA: 146 pts EXE: 29 pts LTZ: 74 pts	Median: 1.2 years	Arm 1 (122 pts): oral O3-FAs 3.3 g daily Arm 2 (127 pts): matching placebo	24 weeks	24 weeks	99%	No differences between the groups both at 12 and 24 weeks in the primary (BPI) and secondary (M-SACRAH, WOMAC and FACT-ES) endpoints
Shen et al. [91] 2018	Exploratory analysis of the study by Hershman [88] in obese pts	110	ANA: 60 pts EXE: 13 pts LTZ: 37 pts	Median: 1.33 years	Arm 1: oral O3-FAs 3.3 g daily Arm 2: matching placebo daily	24 weeks	24 weeks	N.R.	O3-FAs therapy was associated with significant lower BPI scores at 24 weeks vs placebo. Furthermore, a statistically significant improvement in Global Ratings of Change scores for joint pain and stiffness and of M-SACRAH and WOMAC was observed in Arm 1 vs. placebo
Lutsberg et al. [92] 2018	RCT	44	ANA: 31 pts EXE: 1 pt LTZ: 12 pts	Less than 21 days	Arm 1 (22 pts): oral 4.3 g/day of *n*–3 PUFAs Arm 2 (22 pts): matching placebo	24 weeks	24 weeks	86%	Pain severity scores measured by BPI-SF didn’t change significantly by time or treatment arm. A significant difference in quality of life, based on FACT-ES scores, was observed in arm 1 vs. placebo in the short-term (12 weeks)
Henry et al. [93] 2018	RCT	289	N.R.	At least 21 days Mean: 47.9 ± 36.3 weeks	Arm 1 (145 pts): oral Duloxetine 30 mg daily for 1 week, followed by 60 mg daily for 11 weeks, followed by 30 mg daily for another week Arm 2 (144 pts): matching placebo	13 weeks	24 weeks	75%	A greater significant reduction of average joint pain (by BPI-SF) was reported in Arm 1 vs placebo at 12 weeks, but not at 24 weeks. Furthermore, a significant improvement of WOMAC, M-SACRAH and FACT-ES was observed in the Duloxetine arm
Henry et al. [94] 2019	Exploratory analysis of the study by Henry et al. [95] on the basis of BMI categories	289	N.R.	Mean: 47.9 ± 36.3 weeks	Arm 1 (145 pts, of whose 78 obese): oral Duloxetine 30 mg daily for 1 week, followed by 60 mg daily for 11 weeks, followed by 30 mg daily for another week Arm 2 (144 pts, of whose 78 obese): matching placebo	13 weeks	24 weeks	75%	The reduction of pain measured by BPI-SF, was more pronounced in obese patients treated with Duloxetine vs placebo at 12 weeks, while it was similar to placebo in the non-obese group. Similar findings were reported for M-SACRAH, WOMAC, FACT-ES
Kubo et al. [96] 2012	Prospective not controlled study	27	ANA:25 pts LTZ: 2 pts	Mean: 16 months	Arm 1: 5 mg of oral Prednisolone once a day for one week	1 week	2 months	100%	Joint pain symptoms, measured by VAS, improved in 67% of pts immediately after Prednisolone use, with persistent effect at one month in 63% and at 2 months in 52%
Greenlee et al. [97] 2013	Prospective not controlled study	53	ANA: 35 pts EXE: 3 pts LTZ:2 pts	At least 3 months	Arm 1: 2 capsulesx3 times/day or 3 capsulesx2 times/day, each capsule containing 250 mg Glucosamine sulfate potassium chloride and 200 mg Chondroitin sulfate sodium	24 weeks	24 weeks	69.8%	At week 24, 46.2% of pts met the OMERACT-OARSI criteria for self-reported improvements in pain and function, as measured by BPI, WOMAC and M-SACRAH
Campbell et al. [98] 2017	Prospective not controlled study	41	N.R.	At least 14 days	Arm 1: 2500 mcg of sublingual vitB12 daily	3 months	3 months	87.8%	After 3 months, a 23% relative improvement from baseline in worst pain score (by BPI-SF) and 34% in average pain score (BPI-SF) was found. Also, FACT-ES score significantly improved
Alhanafy et al. [99] 2018	Prospective not controlled study	50	N.R.	<1 year: 12 pts 1–3 years: 29 pts >3 years: 9 pts	Arm 1: oral combination of Frusemide 20 mg/Spironolactone 50 mg once a day	4 weeks	4 weeks	92%	All WOMAC sub-scores and quick DASH score significantly improved at the end of the treatment vs. baseline
Santa-Maria et al. [100] 2018	Prospective not controlled study	59	LTZ	Letrozole was started 1–2 weeks following the initial dose of zolendronic acid	Arm 1: 4 mg of i.v. zolendronic acid at baseline and at 6 months	6 months	12 months	88%	A significantly lower incidence of AIA (defined as an increase of 0.22 in HAQ-II and/or an increase of 2 cm in a VAS 0–10) after 1 year was shown in patients receiving zoledronic acid, compared with historical controls from the ELPh trial

Abbreviations: Pts: patients; no: number; AIs: Aromatase Inhibitors; LTZ: Letrozole; 25OHD: 25-hydroxi-Vitamin D; Vit: Vitamin; HAQ-II: Health Assessment Questionnaire; BFI: Brief Fatigue Inventory; MEN-QOL: Menopause Quality Of Life; N.R.: Not Reported; VAS: Visual Analogue Scale; RCT: Randomized Controlled Trial; ANA: Anastrozole; FIQ: Fibromyalgia Impact Questionnaire; BPI-SF: Brief Pain Inventory-Short Form; EXE: Exemestane; SD: Standard Deviation; BCPT-MS: Breast Cancer Prevention Trial Symptom Scale-Musculoskeletal Subscale; PROMIS: Patient-Reported Outcomes Measurement Information System; HGST: HandGrip Strength Test; AUSCAN: Australian/Canadian Osteoarthritis Hand Index; WOMAC: Western Ontario and McMaster Osteoarthritis Index; CPIS: Categorical Pain Intensity Scale; AIA: Aromatase Inhibitors-associated Arthralgia; O3-FAs: Omega-3 Fatty Acids; M-SACRAH: Modified Score for the Assessment and quantification of Chronic Rheumatoid Affections of the Hands; FACT-ES: Functional Assessment of Cancer Therapy-Endocrine System; PUFAs: polyunsaturated fatty acids; BMI: Body Mass Index; OMERACT-OARSI: Outcome Measures in Rheumatology Clinical Trials and Osteoarthritis Research Society International; DASH: Disabilities of the Arm, Shoulder and Hand Score; ELPh trial: Exemestane and Letrozole Pharmacogenetics trial.

**Table 3:** Characteristics of the clinical studies reporting an association between Aromatase Inhibitors (AIs) therapy and autoimmune rheumatic diseases.

Authors	Study Design	Pts (no)	AIs	Time from AIs Therapy and Symptoms Onset	Time from AIs Therapy and Diagnosis	Diagnosis	Autoimmune Laboratory Findings	Treatment for the Rheumatic Disease	Improvement after AIs Discontinuation
Morel et al. [123] 2007	Case report	1	EXE for 4 months	few days	4 months	RA	RF -; anti-CPP -	MTX 15 mg/week	No
Bruzzese et al. [124] 2011	Case report	1	ANA for 4 years	1 year	5 years	RA	RF +; anti-CCP +; Antinuclear ab -; ENA -	MTX 15 mg/week, Methylprednisolone 16 mg/day	No
Bertolini et al. [125] 2011	Case series	3	LTZ for 3 months, followed by EXE for 2 months (1 pt); ANA for 6 months (1 pt); LTZ for 4 months, followed by EXE for one month (1 pt)	Two weeks (1 pt); few weeks (1 pt); 4 months (1 pt)	One year (1 pt); 4 years (1 pt); 3 years (1 pt)	RA (3 pts)	Anti-CCP + (3 pts); RF + (2 pts); Antinuclear ab + 1/160 (2 pts); Antinuclear ab + 1/640 (1 pt)	HCQ 200 mg × 2 times/day (1 pt); SSZ 2 g/day (1 pt); Prednisone 10 mg/day (1 pt)	No (3 pts)
Chao et al. [126] 2009	Case report	1	LTZ for 16 months	16 months	16 months	Accelerated cutaneous nodulosis in pt with RA history	RF+; anti-CCP +	None	Yes (the nodules decreased in size and tenderness)
Scarpa et al. [127] 2011	Descriptive cross-sectional study	18	Type of AIs N.R. Mean duration of the therapy: 12 months	N.R.	N.R.	Undifferentiated SpA (10 pts); oligoarthritis (2 pts); arthralgia (6 pts)	Anti-CCP + (1 pt); RF − (18 pts)	NSAIDs (11 pts), corticosteroids (5 pts), MTX 10 mg/week (3 pts)	Yes (2 pts). N.R. (16 pts)
Laroche et al. [128] 2007	Observational study	24	ANA (20 pts) and LTZ (4 pts); Duration of the therapy: N.R.	2.5 months (mean time)	N.R.	Probable SjS (7 pts); definite SjS (1 pt); RA (1 pt); Hashimoto thyroiditis (1 pt); HCV (2 pts); shoulder tendinitis (1 pt); paraneoplastic aponeurositis (1 pt); OA (2 pts); unknown (7 pts)	Antinuclear ab + >1/160 (9 pts); RF + (4 pts); anti-CCP (2 pts)	NSAIDs (19 pts), Prednisone 10 mg/day for 8 days (9 pts)	N.R.
Guidelli et al. [129] 2012	Case series	3	ANA for 2 years (1 pt); ANA for 3 years (1 pt); LTZ for 3 years (1 pt)	3 months (2 pts); 5 months (1 pt)	1 year (3 pts)	SjS	RF + (2 pts); Antinuclear ab+ 1/320 (2 pts): anti-Ro-SSA + (2 pts); anti-CCP - (3 pts)	N.R.	N.R.
Yasar Bilge et al. [130] 2014	Case report	1	ANA Duration of the therapy: N.R.	N.R.	3 years	SjS and polyneuropathy	RF +; Antinuclear ab+; anti-SSA and SSB -	IVIG treatment (400 mg/kg/day for 5 days monthly for 6 months)	N.R.
Pokhai et al. [131] 2014	Case report	1	LTZ for 4 years, then EXE	2 years	4 years	SS	Antinuclear ab+ 1/1280 with centromeric pattern; anti-centromere B +	N.R.	Yes (an improvement was noted after LTZ discontinuation and substitution with EXE
Mascella et al. [132] 2016	Case report	1	LTZ for 3 months and ANA for one month	3 months	3 months	ASAS	RF+; anti-CCP +; anti-Jo1+; anti-Ro52 +	High dose corticosteroids (Methylprednisolone, 3500 mg bolus injections, followed by 1 mg/kg/day), Azathioprine (100 mg/day)	Yes (a re-exacerbation was described after the resume of another AIs)
Tenti et al. [11] 2019	Case report	1	ANA Duration of the therapy: 6 months	6 months	9 months	APS	Antinuclear ab +; aCL IgG and IgM +; aβ_2_GP1 IgG and IgM+; LAC+	Enoxaparin 6000 IU for 2 times/day, followed by Warfarin, IVIG therapy (400 mg/kg/day for 5 days, followed by 400 mg/kg/day monthly) and HCQ 200 mg × 2 times/day	N.R.

Abbreviations: Pts: patients; no: number; AIs: Aromatase Inhibitors; EXE: Exemestane; RA: Rheumatoid Arthritis; RF: Rheumatoid Factor; anti-CCP: anti-Cyclic Citrullinated Peptide antibodies; MTX: Methotrexate; ANA: Anastrozole; Anti-nuclear ab: Anti-nuclear antibodies; ENA: Extractable Nuclear Antigen; LTZ: Letrozole; HCQ: Hydroxychloroquine; SSZ: Sulfasalazine; N.R.: Not Reported; SpA: SpondyloArthropaty; NSAIDs: Non Steroidal Anti-inflammatory Drugs; SjS: Sjogren’s Syndrome; HCV: Hepatitis C Virus; OA: Osteoarthritis; IVIG: Intravenous Immunoglobulin; SS: Systemic Sclerosis; ASAS: Anti-Synthetase Antibody Syndrome; APS: Anti-Phospholipid Syndrome; aCL: anti-Cardiolipin antibodies; aβ2GPI: anti-β2-GlycoProtein-I antibodies; LAC: Lupus Anti-Coagulant.

**Table 4:** Summary of the studies evaluating the incidence of rheumatic diseases during hormone therapy for breast cancer.

Authors	Country	Study Period	Total Patients	Analyzed Treatment	Reference	Autoimmune Diseases Considered	Incidence Rate Calculation	Estimated Incidence
Chen et al. [147] 2015	U.S.A	1999–2013	238,880	SERM AIs	General population	RA SLE	OR	RA and SERMs: 1.26 for 2–11 months of therapy (95% CI 1.13–1.41); 2.41 for >12 months (95% CI 1.92–3.02;) SLE and SERMs: 1.41 for 2–11 months of therapy (95% CI 1.16–1.71); 2.02 for > 12 months (95% CI 1.29–3.15) RA and AIs: 1.32 for 2–11 months of therapy (95% CI 1.21–1.44); 1.85 for >12 months (95% CI 1.57–2.17). SLE and AIs: 0.84 for 2–11 months of therapy (95% CI 0.70–1.02); 0.77 for >12 months (95% CI 0.50–1.21)
Caprioli et al. [148] 2017	Italy	2004–2013	7533	Tamoxifen AIs	General population	RA	HR and 95% CI	Incident Rate (95% CI) per 1000 person-years Tamoxifen: 3.01 (1.96 to 4.40); AIs: 3.01 (1.96 to 4.40)
Chien et al. [149] 2020	Taiwan	2007–2012	40,761	AIs	Tamoxifen users	Any arthritis (including OA, RA and other arthritis); CTS	HR and 95% CI	AIs and any arthritis HR (95% CI): 1.21 (1.09–1.34) AIs and CTS HR (95% CI): 1.68 (1.22–2.32)
Wadström et al. [150] 2020	Sweden	2006–2016	15,921	Tamoxifen AIs	General population	RA	OR	OR (95% CI): Tamoxifen: 0.86 (0.62 to 1.20) AIs: 0.97 (0.69 to 1.37)

Abbreviations: SERM: Selective Estrogen Receptor Modulator; AIs: Aromatase Inhibitors; RA: Rheumatoid Arthritis; SLE: Systemic Lupus Erythematosus; OR: Odds Ratio; CI: Confidence Interval; HR: Hazard Ratio; OA: Osteoarthritis; CTS: Carpal Tunnel Syndrome.

## Abbreviations

ACOG	American College of Obstetricians and Gynecologists
ACR	American College of Rheumatology
AIA	Aromatase inhibitors-associated arthralgia
AIs	Aromatase inhibitors
Anti-CCP	anti-cyclic citrullinated protein antibodies
aPL	anti-phospholipid antibodies
APS	Antiphospholipid syndrome
ArKO	Aromatase gene knockout
ASAS	Anti-synthetase antibody syndrome
ASCO	American Society of Clinical Oncology
BC	Breast cancer
Bcl-2 protein	B cell lymphoma-2 protein
BMD	Bone mineral density
BMI	Body mass index
BPI-SF	Brief pain inventory—short form
CDK	Cycline dependent kynase
CRP	C reactive protein
CTLs	Cytotoxic-T-lymphocytes
CV	Cardiovascular
DAS	Disease activity score
DASH	Disabilities of the arm, shoulder and hand
DLE	Discoid lupus erythematosus
DXA	Dual-energy X-ray absorptiometry
E1	Estrone
E2	Estradiol
ELPh	Exemestane and letrozole pharmacogenetics
ER	Estrogen receptors
ESMO	European Society of Medical Oncology
ESR	Erythrocyte sedimentation rate
FACT-ES TOI	Functional Assessment of Cancer Therapy-Endocrine Scale Trial Outcome Index
FDA	Food and Drug Administration
GSM	Genitourinary syndrome of menopause
GWAS	Genome-Wide Association Study
HAQII	Health Assessment Questionnaire II
HLA	Human leucocyte antigen
HR	Hazard ratio
IGF	Insulin-like growth factor
IL	Interleukin
IL-17RA	Interleukin-17 receptor A
INF	Interferon
IR	Incident rate
IRF	Interferon regulatory factor
LHRH	Luteinizing hormone-releasing hormone
MCF	Metacarpo-phalangeal
MCP	Monocyte chemoattractant protein
MHC	Major histocompatibility complex
MMP-3	Metalloproteinases-3
MRI	Magnetic resonance imaging
M-SACRAH	Modified score for the assessment and quantification of chronic rheumatoid affection of the hands
NO	Nitric oxide
NSAIDs	Non-steroidal anti-inflammatory drugs
O3-FAs	Omega-3 fatty acids
OA	Osteoarthritis
OMERACT-OARSI	Outcome Measures in Rheumatology Clinical Trials and Osteoarthritis Research Society International
OPG	Osteoprotegerin
PD-1	Programmed cell death receptor
PDL1	PD-1 ligand-1
PG	Prostaglandin
PIP	Proximal inter-phalangeal
PR	Progesterone receptors
RA	Rheumatoid arthritis
RANKL	Receptor activator of nuclear factor-kB ligand
RCTs	Randomized controlled trials
RF	Rheumatoid factor
SERD	Selective estrogen receptor down-regulators
SERM	Selective estrogen receptor modulator
SjS	Sjogren syndrome
SLE	Systemic lupus erythematosus
SNPs	Single-nucleotide polymorphisms
SpA	Spondyloarthropaty
SS	Systemic sclerosis
SSA	Sjogren syndrome-associated autoantigen
TCL1A	T-cell leukemia 1A
TNF	Tumor necrosis factor
US	Ultrasound
VDBP	Vitamin D-binding protein
VDR	Vitamin D receptor
WOMAC	Western Ontario and McMaster Universities Osteoarthritis index
